# The impact of nitroglycerine and volume on gastric tube microperfusion assessed by indocyanine green fluorescence imaging

**DOI:** 10.1038/s41598-022-26545-9

**Published:** 2022-12-27

**Authors:** Philipp H. von Kroge, Detlef Russ, Henrik C. Rieß, Eike S. Debus, Hans. O. Pinnschmidt, Jakob R. Izbicki, Oliver Mann, Sabine H. Wipper, Anna Duprée

**Affiliations:** 1 Department of General, Visceral and Thoracic Surgery, University Medical Center Hamburg-Eppendorf, Hamburg, Germany; 2 Department for the Development of Applications, Institute for Laser Technology, University Ulm, Ulm, Germany; 3 Department of Vascular Medicine, University Heart Center, University Medical Center Hamburg-Eppendorf, Hamburg, Germany; 4 Department of Medical Biometry and Epidemiology, University Medical Center Hamburg-Eppendorf, Hamburg, Germany; 5 Department of Vascular Surgery, University Medical Center Innsbruck, Innsbruck, Austria

**Keywords:** Oesophageal cancer, Risk factors, Gastrointestinal models

## Abstract

The influence of hypervolemia and intraoperative administration of nitroglycerine on gastric tube microperfusion remains unclear The present study aimed to investigate the impact of different hemodynamic settings on gastric tube microperfusion quantified by fluorescence imaging with Indocyanine green (ICG-FI) as a promising tool for perfusion evaluation. Three groups with seven pigs each were formed using noradrenaline, nitroglycerin, and hypervolemia for hemodynamic management, respectively. ICG-FI, hemodynamic parameters, and transit-time flow measurement (TTFM) in the right gastroepiploic artery were continuously assessed. Fluorescent microspheres (FM) were administered, and the partial pressure of tissue oxygen was quantified. The administration of nitroglycerine and hypervolemia were both associated with significantly impaired microperfusion compared to the noradrenaline group quantified by ICG-FI. Even the most minor differences in microperfusion could be sufficiently predicted which, however, could not be represented by the mean arterial pressure measurement. Histopathological findings supported these results with a higher degree of epithelial damage in areas with impaired perfusion. The values measured by ICG-FI significantly correlated with the FM measurement. Using tissue oxygenation and TTFM for perfusion measurement, changes in microperfusion could not be comprehended. Our results support current clinical practice with restrictive volume and catecholamine administration in major surgery. Hypervolemia and continuous administration of nitroglycerine should be avoided.

## Introduction

Esophagectomy with reconstruction via gastric tube pull-up remains the most successful treatment in non-metastatic esophageal carcinoma^1^. However, the formation of gastroesophageal anastomosis can be complicated, resulting in several life-threatening events such as leakage with mediastinitis, sepsis, and lethal outcomes^2–4^. Leakage occurrence has been associated with reduced microvascular blood flow and consecutive hypoxemia^5,6^. Due to the surgical technique of gastric tube formation with ligation of all gastric vessels except the right gastroepiploic artery and its arcade, a decrease in microperfusion can be observed mainly in the upper part of the gastric tube^7,8^.

Up to now, several strategies to improve the gastric tube microperfusion^9–12^ or to reduce venous congestion have been reported^13–15^. However, there is increasing evidence that excessive intravenous fluid administration has a relevant impact on developing anastomotic complications ^16^.

Indocyanine green fluorescence imaging (ICG-FI) is a promising tool for assessing and quantifying tissue microperfusion. Several retrospective clinical studies indicate a benefit of the technology in terms of anastomotic leakage rate^17–19^. Recently, we validated the ICG-FI by assessing gastric tube perfusion in a porcine model. Perfusion could be predicted by using the slope of fluorescence intensity (SFI), background subtracted peak fluorescence intensity (BSFI), and time to slope (TTP) for quantification. ICG-FI was compared with fluorescent microspheres (FM); the experimental gold standard for perfusion quantification^7^. In addition, we evaluated the parameters in a clinical setting in patients undergoing esophagectomy with gastric tube creation. A SFI reduction of up to 32% and a BSFI reduction of up to 23% in the anastomotic region were not associated with anastomotic leakage^20^.

In the present study, we aimed to detect changes in microperfusion of gastric tube formation by using ICG-FI to assess the microperfusion. We hypothesized that the application of nitroglycerine leads to improved microperfusion due to reduced venous congestion, while excessive hemodilution results in an impaired microperfusion caused by edema^15,16^.

## Methods

All experiments were performed in compliance with the Institutional Review Board for the care of animals following with the National Institutes of Health guidelines for ethical animal research. The protocol was authorized by the Committee on the Ethics of Animal Experiments of the Authority for Health and Consumer Protection Hamburg (protocol number: 113/14).

The following methods regarding anesthesia, surgical procedures, fluorescent microspheres, fluorescence intensity, and image and data analysis have been reported in our group’s previous publication.

### Experimental protocol

The experiments were performed on 21 pigs of either sex weighing 56.0 ± 2.6 kg at the Institute for Surgical Research at the University Center Hamburg-Eppendorf (Hamburg, Germany).

After intramuscular premedication with azaperone (4 mg/kg), midazolam (0.3 mg/kg), ketamine (5 mg/kg), and atropine sulfate (0.15 mg/kg), intravenous anesthesia was induced by propofol (0.06 mg/kg) and maintained by continuous infusion of fentanyl (0.01 mg/kg/h), midazolam (0.1 mg/kg/h), ketamine (0.1 mg/kg/h), and propofol (3 mg/kg/h). The animals were endotracheal intubated and pressure-controlled ventilated at 15 cm H_2_O with a positive end-expiratory pressure of 7 cm H_2_O at 16 breaths per minute using 30% oxygen. Heparin (400 U/kg) was administered to achieve an activated clotting time of at least 300 s with repetitive administrations every 3 h^7^.

A 6 F arterial catheter was introduced to the right carotid artery for permanent assessment of heart frequency (HF), and median arterial blood pressure (MAP), as well as a central venous catheter into the right jugular vein for delivery of infusions, heparin, and ICG as well as the measurement of central venous pressure (CVP). A 4F arterial catheter with an embedded thermistor was inserted into the right femoral artery for continuous hemodynamic monitoring of stroke volume, cardiac output, systemic vascular resistance (SVR), and global end-diastolic volume (GEDV) by the determination of thermodilution. These parameters were documented by the PiCCO device (Pulsion Medical Systems, Munich, Germany) based on arterial pulse contour analysis. For the FM application, a catheter was placed in the left atrium. A pigtail catheter was inserted into the abdominal aorta via the right femoral artery to withdraw reference blood samples with a known constant withdrawal rate to calculate the flow rate per tissue sample^7^.

**Figure 1:**
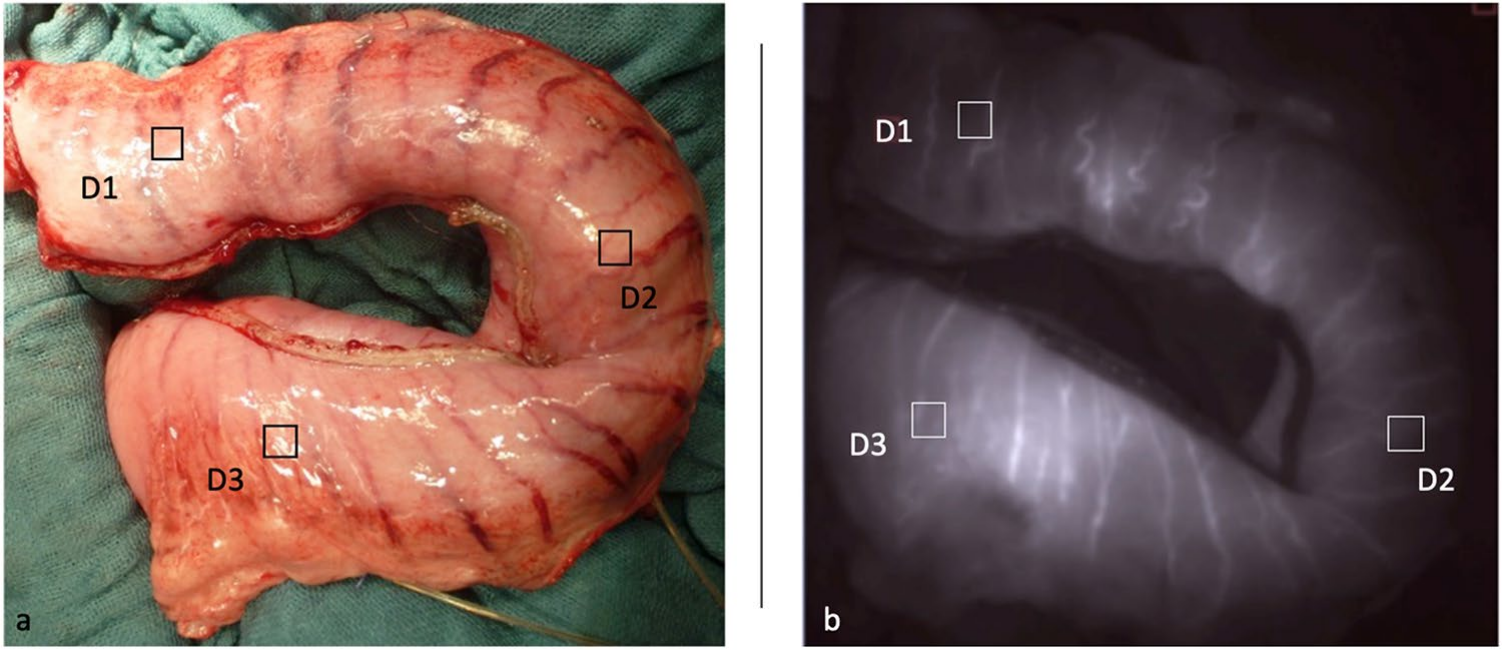
Macroscopic and fluorescent image of the gastric tube. The left picture (**a**) shows the macroscopic picture of the gastric tube. Three regions of interest (ROI) are marked: The prepyloric region (D3), the corpus (D2), and the tip (D1). The right picture (**b**) shows the associated fluorescence angiographic image of the gastric tube with the same marked ROIs.

First, a midline laparotomy was performed. A catheter was placed into the bladder for urinary drainage. The gastric tube formation was performed under ligation of all arteries despite the gastroepiploic artery and its arcade. The dissection of the minor curvature was performed using Endo-GIA (Covidien, black cartridge), resulting in a gastric tube with a three-centimeter diameter. In this way, the perfusion depended on a single vessel leading to a standardized “one-vessel-model”. Three regions of interest (ROI) were defined: fundus (D1), corpus (D2), and prepyloric (D3). Figure 1 shows a macroscopic and an associated fluorescence angiographic image of the gastric tube with marked ROIs in the defined areas. After that, a flexible polarographic measuring probe (Licox, Mielkendorf, Germany) for continuous measurement of the tissue oxygen tension (partial pressure of tissue oxygen [tpO_2_]) was inserted in all defined areas of the tube. For the continuous determination of blood flow of the right gastroepiploic artery, a transient time flow measurement (TTFM) probe (CardioMed Flowmeter, Medi-Stim AS, Oslo, Norway) was placed around the vessel. The abdomen was covered partially to reduce loss of fluid and temperature^7^.

**Figure 2:**
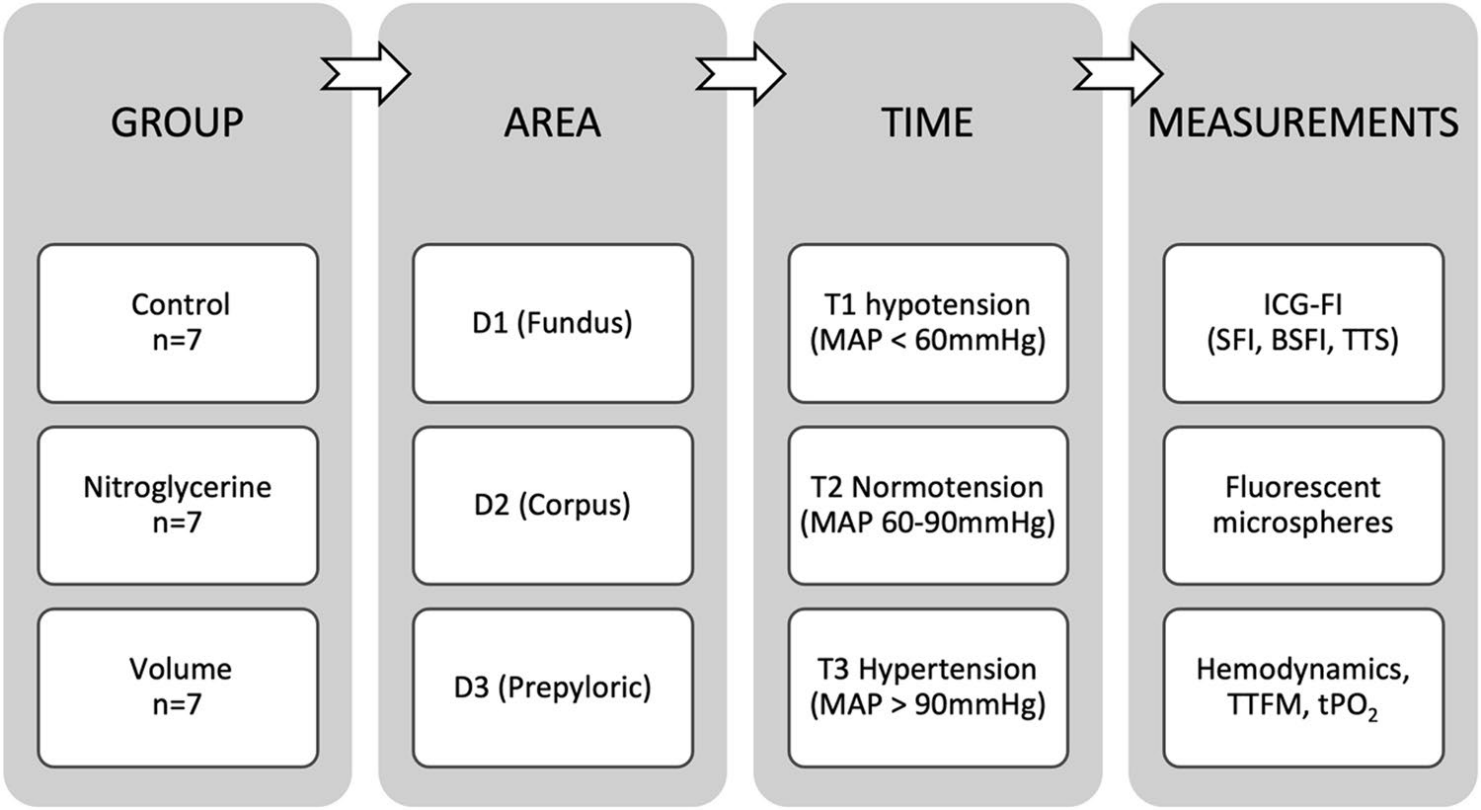
Experimental setting. Three groups were formed from a total of 21 animals: A control group (n = 7), a group with continuous infusion of nitroglycerine (n = 7), and a group with hypervolemia. In each animal, a gastric tube was created and divided into three segments: Gastric fundus (D1), gastric corpus (D2), and prepyloric region (D3). Various measurements were carried out at three predefined times (T1: hypotension, T2: normotension; T3: hypertension): Indocyanine green fluorescence imaging (ICG-FI) with the calculation of slope of fluorescence intensity (SFI), background subtracted fluorescence intensity (BSFI) and time to slope (TTS), application of fluorescent microspheres, continuous measurement of the hemodynamics, transit time flow measurement (TTFM) of the right gastroepiploic arteria and tissue oxygenation (tPO_2_). *MAP* mean arterial blood pressure.

Baseline measurements were done after completing the preparation. The hemodynamic, metabolic, and tissue oxygenation parameters were determined after blood pressure modulation. Mean arterial blood pressure (MAP) was modified by using norepinephrine and propofol, resulting in 3 measurements: hypotension (MAP < 60 mmHg, T1), normotension (MAP 60–90 mmHg, T2), and hypertension (MAP > 90 mmHg, T3)^7^. Animals were randomly assigned to the different treatment groups: Control (control; n = 7), Nitroglycerine (Nitro; n = 7), and hypervolemia (Vol; n = 7). As the administration of catecholamines represents the standard of hemodynamic management in clinical praxis, calculated values of the control group represent the reference values when comparing the different groups. Nitroglycerin was applicated by continuous intravenous administration of nitroglycerin (3.5 µg/kg/min). Hypervolemia was defined by a global end-diastolic volume (GEDV) > 700 ml and at least 5 L of crystalloids and colloids (ratio 2:1). Every time the target MAP was reached, a 30-min stabilization period was respected. ICG-FI and FM were performed at each measurement. Hemodynamic parameters, TTFM, and tPO_2,_ were captured for each measurement^7^. After finishing the measures, the animals were euthanized by a veterinarian using T 61 under deep anesthesia. The experimental setting is shown in simplified form in Fig. 2.

### Fluorescent microspheres

FMs of 15 μm in diameter (Molecular Probes, Eugene, Ore) were used to measure the median blood flow (ml/min/g) at each measurement. Microspheres labeled with different fluorescent colors were randomly selected for the application. Excitation and emission wavelengths for each of the fluorescence microspheres were as follows: blue, 356/424 nm; blue-green, 427/468 nm; yellow-green, 495/505 nm; orange, 534/554 nm; red, 570/598 nm; crimson, 612/638 nm; and scarlet, 651/680 nm. They did not interfere with the excitation and emission wavelength of ICG. Each vial of FMs was placed in an ultrasonic water bath for 5–10 min to disperse the microspheres and vortexed twice for 3 min to ensure proper mixing before injection. Approximately 3.33 × 10^6^ FMs were suspended in physiological saline solution to a volume of 10 mL and constantly injected over 60 s into the left atrium at each measurement. Reference blood samples were withdrawn in anticoagulated (5 mL of 3.13% sodium citrate) syringes with a constant-rate withdrawal pump at 3.18 mL/min over 3 min. Injection of FMs was started when the withdrawn blood reached the suction syringe. At the end of the experiments, the gastric tube was excised and fixed in 10% formaldehyde for 6–8 days. Afterwards it was dissected into 28 tissue pieces with a mean weight of 3.5 ± 0.2 g^7^. According to the standard method published by Glenny et al., the tissue pieces were processed for blood flow determination using spectrofluorometry^21^.

### Fluorescence intensity (FI)

Perfusion was assessed with the FI-system (LLS GmbH, Ulm, Germany). ICG was administered intravenously through a central venous line to ensure adequate mixing. The dose was adapted to the body weight (0.02 mg/kg body weight). The bowel was illuminated with near-infrared light at a wavelength of 785 nm provided by infrared laser diodes with a total output of 80 mW in a field of view of 10 cm in diameter. The fluorescence emission of the excited dye was detected by an infrared-sensitive charged-couple device camera system equipped with a band-pass filter for the selective transmission of light at the central wavelength of 830 nm. The dynamic range of the camera was 54 dB. The camera`s signals were digitized with a frame grabber card that provides a resolution of 8 bits. Images were acquired at a rate of 25 frames per second and were recorded in real-time. The charge-coupled device imaging system was positioned on the exposed surface of the gastric tube at a distance of ≈ 25 cm. The camera distance was measured and calibrated after each measurement. The FI was displayed in real-time on a computer monitor and analyzed using a digital image processing system with a temporal resolution of 20 ms and spatial resolution of ≈ 0.2 mm at a penetration depth of 4 mm^7^.

### Image and data analysis

The analysis of fluorescence data has already been described in detail before^22^. Briefly, to obtain a quantitative measure for the fluorescence intensity, we calculated both the mean value and the SD of the measured pixel intensities in the region of interest on the tissue wall for each image in a sequence of 60 s after the injection of the ICG. This region of interest was 30 × 30 pixels, corresponding to an area of ≈ 8 × 8 mm on the gastric surface, depending on the distance from the camera. To measure gastric perfusion, we calculated three different parameters derived from the time-dependent fluorescence signal^7^.

- *Background-Subtracted Peak Fluorescence Intensity (BSFI)* To calculate BSFI from the time-dependent fluorescence intensity, the initial intensity value before the injection of ICG was subtracted from the peak fluorescence intensity during the first passage of the dye through the gastric tube^22^.
- *The slope of Fluorescence Intensity (SFI)* This parameter represents by the maximal slope during the increase of the time-dependent fluorescence intensity induced by the first wave of the dye, which passes the capillaries of the gastric tissue^22^.
- *Time to slope (TTS)* TTS is defined as the time between injection of the ICG and the first fluorescent signal at the ROI. TTS of areas D2 and D1 were placed in relation to area D3 as a TTS ratio^7^.

**Figure 3:**
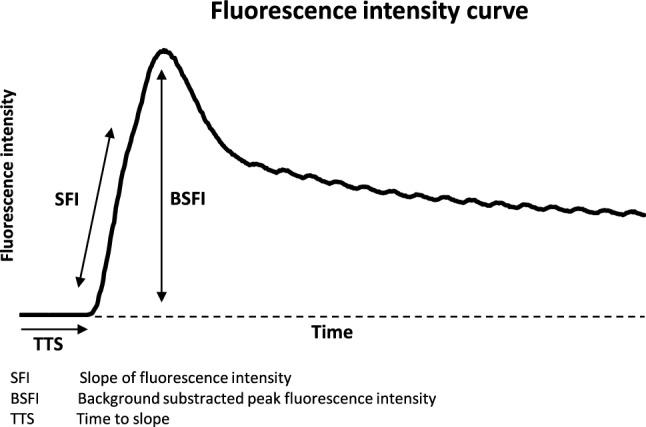
Figure shows a time-dependent fluorescence intensity curve and a schematic presentation for calculating the quantification parameters SFI, BSFI, and TTS. TTS defines the time between Indocyanine green (ICG) administration and the first fluorescence signal. SFI represents the maximal slope of the curve. For the calculation of BSFI, the initial fluorescence intensity before injection of ICG is subtracted from the peak fluorescence intensity.

Each ICG-FI sequence was recorded online for 120 s with real-time digitizing. BSFI, SFI, and TTS data were analyzed offline directly after the images were recorded with a customized software package “Meteroarchive VCL LLS Fluoreszenzangiographie V 1.0” (LLS GmbH, Ulm, Germany)^7^. A scheme of the calculation of the parameters is shown in Fig. 3.

### Histopathology

Representative specimens of the gastric tube ROIs were assessed and stored in 3.5% buffered formalin. Afterwards, all samples were routinely processed and embedded in paraffin, and 5-µm slices were stained with hematoxylin and eosin^7^. The slices were examined by an experienced pathologist (blinded to the treatment) using an established scoring system^23^. Mucosa taken from the anterior gastric corpus was divided into the following grades under a light microscope: grade 0: normal gastric mucosa; grade 1: surface mucosa cells were damaged; grade 2: in addition to extensive luminal damage, cells lining the gastric pits were also disrupted and exfoliated; grade 3: cell destruction extended into the gastric gland^23^.

### Statistics

Data from all animals were expressed as mean ± SD. Comparisons of hemodynamics, TTFM, tPO_2,_ FM, and ICG-FI data among the hemodynamic states were performed by ANOVA. Data for SFIcor, BSFIcor, TTFM, and tissue oxygenation were evaluated by using means of boxplots. Data values of the variables SFI, BSFI, and TTS were ln-transformed because their data distributions were right-skewed. The ln-transformed data of SFI, BSFI, and TTS were then as dependent variables subjected to general linear mixed model analyses (SPSS routine GENLINMIXED), assuming random intercepts for animal-by-rater and time points as repeated measures within animal-by-rater-by-area. Group, area, time point, FM (log_2_-transformed), and all of their 2-way-interaction terms were considered as fixed effects. Interaction terms were removed from the model if they were not significant as judged based on their F values. Model-estimated marginal means were obtained and pairwise comparisons of group, area, and time point were made. A value of p < 0.05 was considered statistically significant. Statistical analysis was performed with the SPSS statistical software package 27.0 (SPSS Inc, Chicago, Ill).

## Results

ICG-FI images of the gastric tube perfusion were obtained in all 21 animals. Systemic hemodynamic parameters were recorded continuously and are summarized in Table 1. The predefined MAP level could be achieved in all groups.

**Table 1:** Hemodynamic parameters.

Group	Time	MAP (mmHg)	HF (bpm)	CVD (cm H_2_O)	GEDV (mL)	SVR (dyn. sec/cm^5^)
1 (control)	T1	51.6 $\pm$ 4.6	106.3 $\pm$ 31.3	7.0 $\pm$ 1.4	634.0 $\pm$ 64.2	677.1 $\pm$ 124.5
	T2	79.6 $\pm$ 5.9	136.1 $\pm$ 24.6	6.1 $\pm$ 1.5	651.1 $\pm$ 119.3	1104.3 $\pm$ 228.6
	T3	101.6 $\pm$ 10.1	138.7 $\pm$ 21.3	5.6 $\pm$ 1.4	637.0 $\pm$ 129.7	1585.9 $\pm$ 450.0
2 (nitroglycerine)	T1	52.7 $\pm$ 6.0	87.1 $\pm$ 21.4	4.6 $\pm$ 2.2	694.1 $\pm$ 114.4	988.0 $\pm$ 481.0
	T2	81.4 $\pm$ 7.3	108.4 $\pm$ 19.5	4.1 $\pm$ 1.9	670.4 $\pm$ 78.4	1598.6 $\pm$ 848.4
	T3	102.7 $\pm$ 2.4	110.4 $\pm$ 24.2	5.6 $\pm$ 2.2	659.1 $\pm$ 91.3	2734.3 $\pm$ 1395.7
3 (volume)	T1	52.6 $\pm$ 5.0	53.1 $\pm$ 15.5	11.3 $\pm$ 1.3	883.0 $\pm$ 127.2	871.4 $\pm$ 264.4
	T2	83.1 $\pm$ 2.0	67.1 $\pm$ 15.3	10.4 $\pm$ 0.8	905.5 $\pm$ 190.1	1405.7 $\pm$ 262.0
	T3	104.2 $\pm$ 8.3	75.5 $\pm$ 15.1	10.2 $\pm$ 1.2	794.3 $\pm$ 162.1	1857.5 $\pm$ 270.1

*MAP* mean arterial pressure, *HR* heart rate, *CVP* central venous pressure, *GEDV* global end-diastolic volume, *SVR* systemic vascular resistance, *T1* hypotension (MAP < 60 mmHg); *T2* normotension (MAP 60–90 mmHg), *T3* hypertension (MAP > 90 mmHg). Hemodynamics were stable through all measurements in all groups. GEDV and CVD were significantly higher in Group 3 (p < 0.05). CVD was significantly lower in group 2 compared to groups 1 and 3 (p < 0.05). MAP was significantly different between all measurement times (p < 0.05).

### Assessment of organ perfusion by ICG-FI

Gastric tube perfusion was quantified by calculating SFI, BSFI, and TTS from the time-dependent fluorescence intensity curve.

### SFI

**Figure 4:**
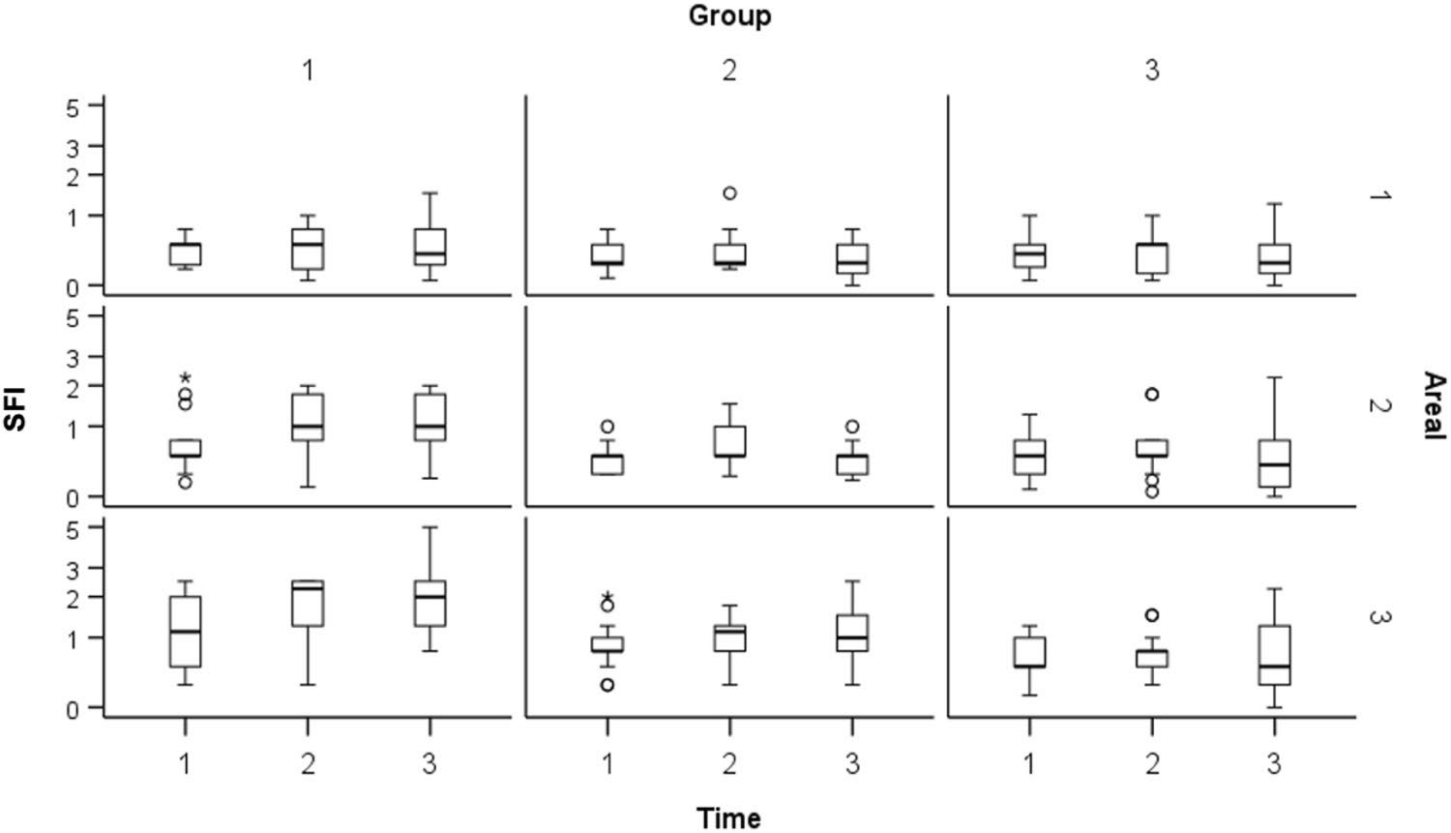
Slope of fluorescence intensity (SFI) values of all groups (1 = control, 2 = nitroglycerine, 3 = volume) are shown in logarithmic form in boxplots at each measurement time (1 = Hypotension, 2 = Normotension, 3 = Hypertension) in each areal (1 = tip, 2 = corpus, 3 = prepyloric). Circles (°) represent mild outliers and asterisks (*) represent extreme outliers. The y-axis shows a logarithmic representation of the SFI values while the other axes represent categorical variables. SFI represents the maximal increase of the time-dependent fluorescence intensity induced by the first wave of the Indocyanine green (ICG), which passes the capillaries of the gastric tissue. SFI-values in groups two and three were significantly lower in areal 2 and 3 compared to the control group (p < 0.001). SFI-values in areal 1 showed no significant differences between all groups. In group 3, hypertension resulted in decreasing SFI indicating impaired perfusion.

SFI-values in the control group indicate significantly impaired perfusion in the distal area (D1) of the gastric tube (T1: 0.43 ± 0.22; T2: 0.52 ± 0.35; T3: 0.52 ± 0.43) while the pyloric region (D3) shows enhanced FI during all measurement (T1: 1.29 ± 0.8; T2: 1.86 ± 0.76; T3: 2.16 ± 1.66). Perfusion level in D2 (T1: 0.76 ± 0.62; T2: 1.13 ± 0.55; T3: 1.10 ± 0.57) was significantly lower compared to the pyloric region (p < 0.001). SFI-values in group 2 were significantly lower in areas D2 and D3 compared to the control group (p < 0.001). SFI-values in area D1 showed no significant differences. Increasing MAP resulted in higher SFI-values in the pyloric region (D3 T1: 0.91 ± 0.50; T2: 1.09 ± 0.43; T3: 1.20 ± 0.63). Area D1 and D2 showed highest SFI-values at normotension; hypertension resulted in decreasing SFI (D1 T1: 0.35 ± 0.22; T2: 0.42 ± 0.35; T3: 0.32 ± 0.24; D2 T1: 0.50 ± 0.22; T2: 0.64 ± 0.37; T3: 0.44 ± 0.23). In group 3 SFI values were significantly lower in areas D2 and D3 compared to the control group at all measurement time points (p < 0.05). SFI-values in area D1 showed no significant differences. Area D3 showed slightly higher values with increasing MAP (T1: 0.63 ± 0.33; T2: 0.77 ± 0.37; T3: 0.75 ± 0.69). Areas D1 and D2 showed highest SFI-values at normotension; hypertension resulted in decreasing SFI (D1 T1: 0.41 ± 0.29; T2: 0.41 ± 0.30; T3: 0.33 ± 0.34; D2 T1: 0.56 ± 0.39; T2: 0.64 ± 0.51; T3: 0.55 ± 0.61). There was no significant difference in area D1 across all groups. Area D2 showed significantly decreased SFI values in groups 2 and 3 compared to the control group. Area D3 showed significantly different SFI values across all groups (p < 0.001). SFI-values are represented graphically in Fig. 4.

### BSFI

**Figure 5:**
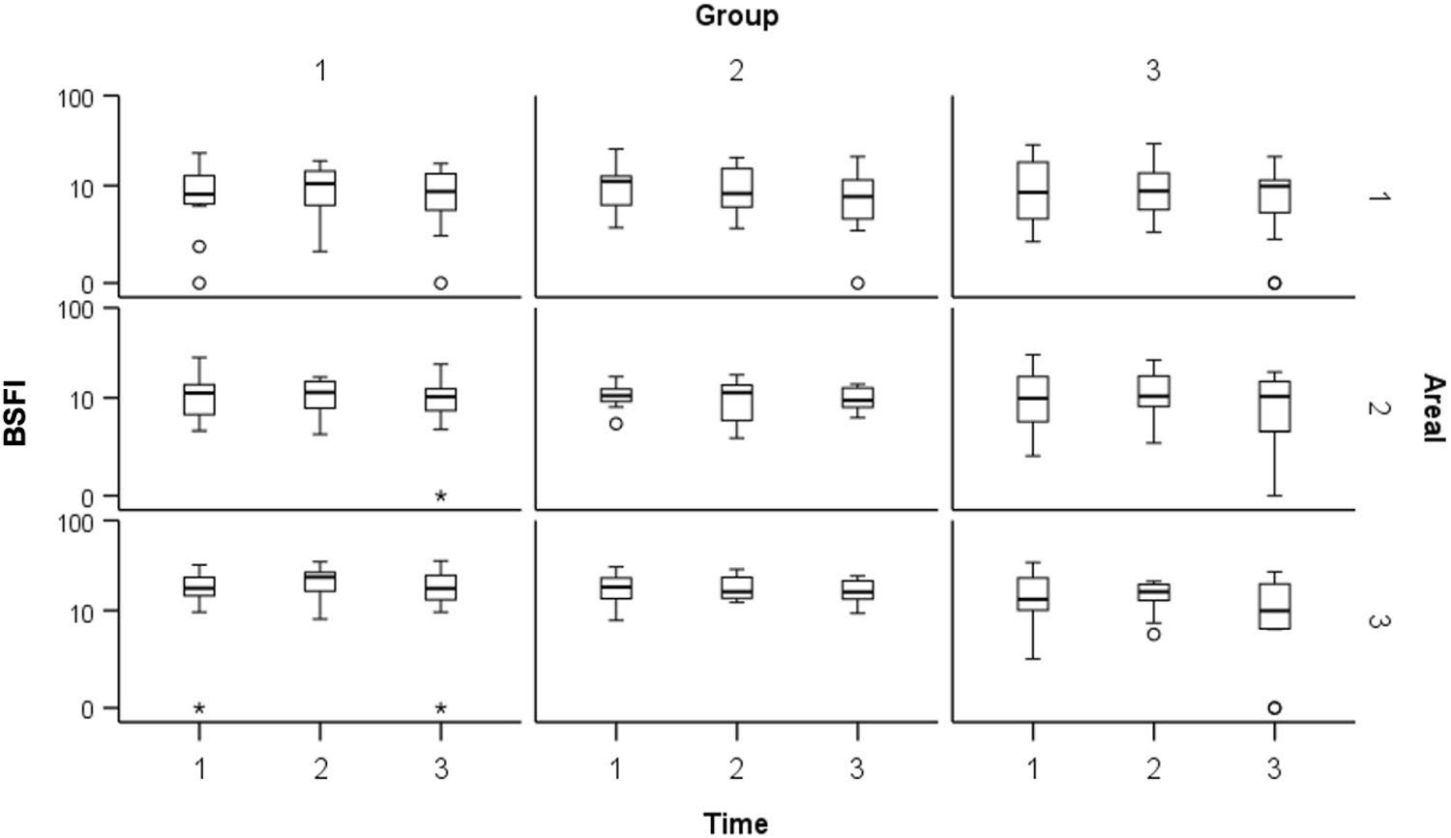
Background subtracted peak fluorescence intensity (BSFI) values of all groups (1 = control, 2 = nitroglycerine, 3 = volume) are shown in logarithmic form in boxplots at each measurement time (1 = Hypotension, 2 = Normotension, 3 = Hypertension) in each areal (1 = tip, 2 = corpus, 3 = prepyloric). Circles (°) represent mild outliers and asterisks (*) represent extreme outliers. The y-axis shows a logarithmic representation of the BSFI values while the other axes represent categorical variables. BSFI represents the difference in the initial intensity value before the injection of ICG was subtracted from the peak fluorescence intensity during the first passage of the dye through the gastric tube. Across all groups, areal 3 showed lower BSFI-values in group 3 compared to group 1 (p < 0.05). Group 2 showed similar trends, which were not statistically significant.

In group 1, BSFI was significantly increased during all measurements in area D3 (T1: 18.2 ± 8.3; T2: 22.8 ± 7.8; T3: 18.8 ± 9.5) compared to area D2 (T1: 12.0 ± 6.8; T2: 11.4 ± 4.4; T3: 10.6 ± 6.2) and area D1 (T1: 9.3 ± 6.1; T2: 10.6 ± 5.9; T3: 9.2 ± 5.6), respectively (p < 0.05). Area D1 and D2 showed no significant difference. Group 2 showed similar results compared to the control group with significantly higher values D3 (T1: 18.8 ± 6.7; T2: 19.1 ± 6.0; T3: 17.1 ± 4.8) compared to area D2 (T1: 11.2 ± 3.4; T2: 10.9 ± 5.0; T3: 10.1 ± 3.0) and area D1 (T1: 10.6 ± 6.0; T2: 10.0 ± 5.7; T3: 8.1 ± 6.1), respectively. There were no significant differences between areas D1 and D2. Likewise, in group 3 area D3 (T1: 18.8 ± 6.7; T2: 19.1 ± 6.0; T3: 17.1 ± 4.8) showed significantly higher values compared to area D2 (T1: 11.2 ± 3.4; T2: 10.9 ± 5.0; T3: 10.1 ± 3.0) and area D1 (T1: 10.6 ± 6.0; T2: 10.0 ± 5.7; T3: 8.1 ± 6.1), respectively. Area D1 and D2 also showed no significant differences. Across all groups, area D3 showed lower BSFI-values in group 3 compared to group 1 (p < 0.05). The difference between groups 2 and 3 showed similar trends, but were not statistically significant (p = 0.053). BSFI-values of all groups are represented graphically Fig. 5.

### TTS

The TTS-ratio showed a delayed increase of fluorescence intensity in the areas D1 and D2 compared to the prepyloric region in all groups. In group 1, TTS-ratio was significantly prolonged in area D1 at all measurements (T1: 2.47 ± 1.81, T2: 1.77 ± 0.58; T3: 4.48 ± 1.94) compared to D2. The values in area D2 (T1: 1.47 ± 0.51; T2: 1.76 ± 1.39; T3: 1.41 ± 0.40) didn’t show any significant differences at the various measurements. In group 2 there were no statistically significant changes of the TTS-ratio in area D1 (T1: 1.72 ± 0.49, T2: 1.67 ± 0.44, 1.75 ± 0.67) and area D2 (T1: 1.41 ± 0.40, T2: 1.38 ± 0.44, T3: 1.27 ± 0.25), respectively. In group 3, TTS-radio in D1 was slightly higher at normotension (1.49 ± 0.53) compared to T1 and T2 (1.36 ± 0.32 and 1.39 ± 0.32, respectively). TTS-ratio in area D2 showed similar trends (T1: 1.15 ± 0.14, T2: 1.19 ± 0.22, T3: 1.14 ± 0.30).

### TTFM-flow

**Figure 6:**
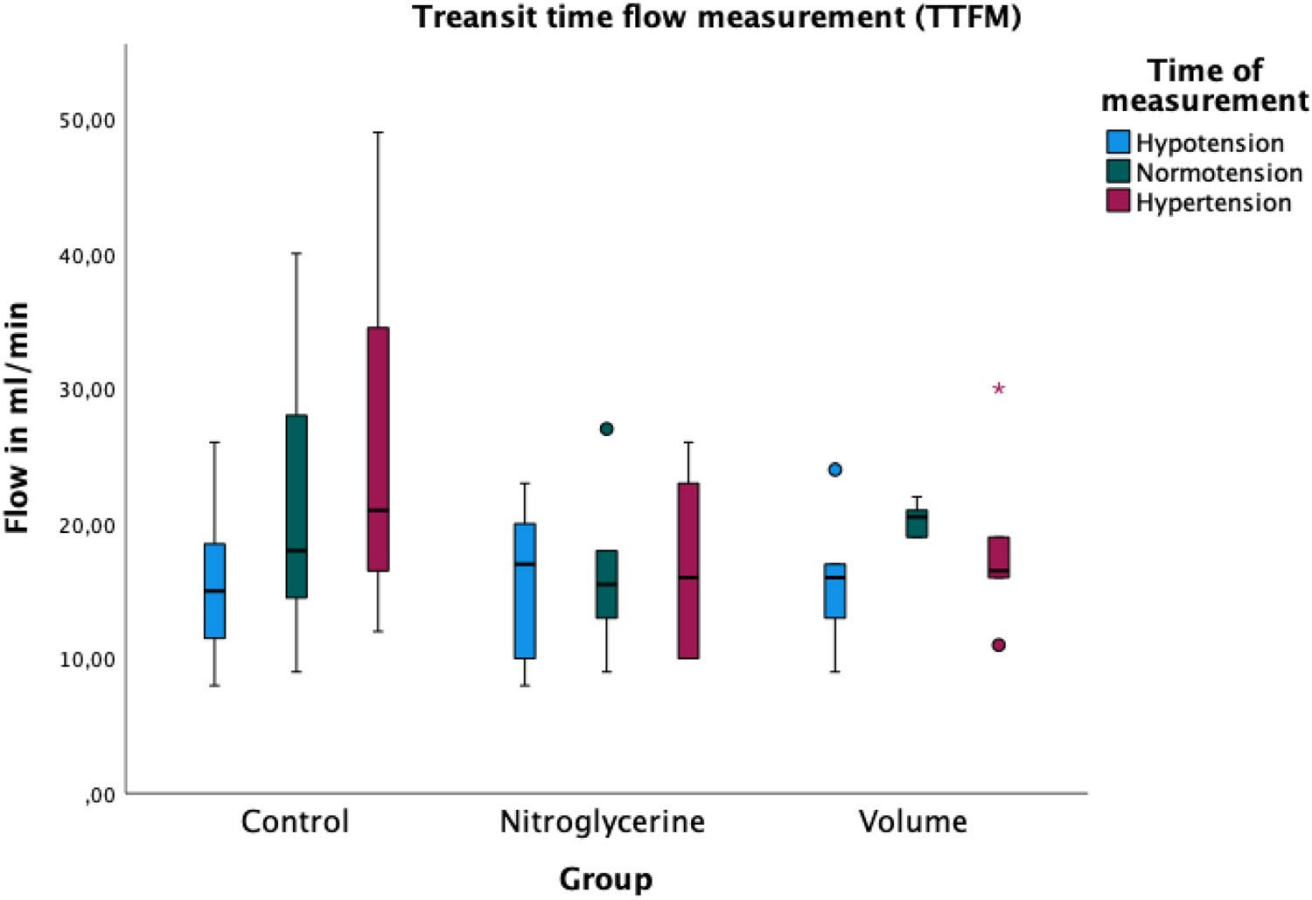
Transit time flow measurement (TTFM) at the right gastroepiploic artery shown as boxplots. Circles (°) represent mild outliers and asterisks (*) represent extreme outliers. The flow is increasing with increasing median arterial blood pressure. The substitution of nitroglycerine is not associated with changes in flow. Hypervolemia shows improved flow at normotension compared to hypo- and hypertension. There were no significant differences between the groups and the time of measurement.

Flow in the gastro-epiploic artery measured by TTFM is shown in Fig. 6. The control group showed an increasing flow with rising MAP. Hypervolemia also resulted in increasing flow at normotension while hypertension showed decreasing flow. Substitution of nitroglycerin in group 2 resulted in no artery flow changes. There were no significant differences between the groups and the time points of measurement.

### Tissue oxygenation

**Figure 7:**
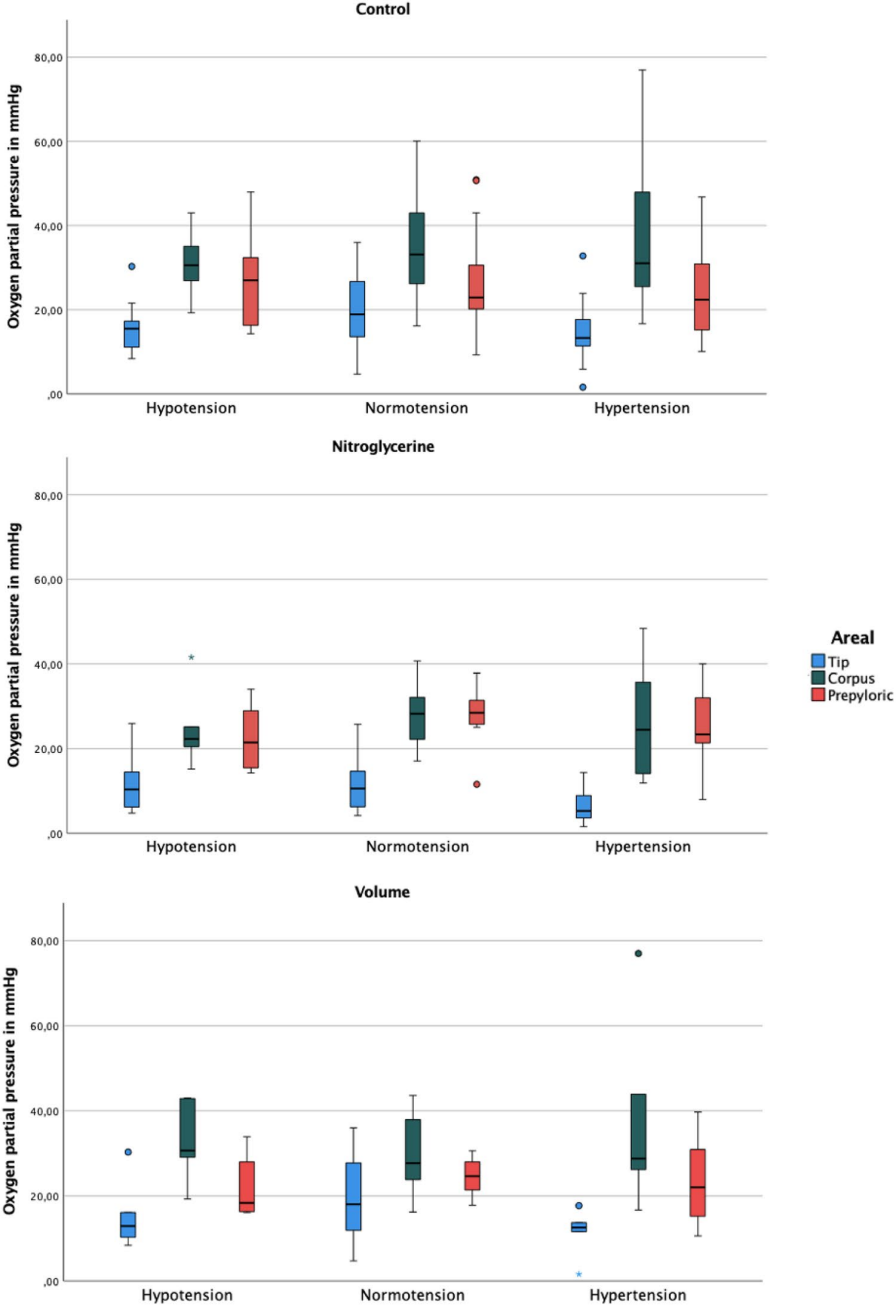
Tissue oxygenation of all groups shown as boxplots. Circles (°) represent mild outliers and asterisks (*) represent extreme outliers. In all groups, the tip shows significantly reduced tissue oxygen partial pressure. In the control group, tissue oxygenation is highest in the corpus. Administration of nitroglycerine results in no significant differences between corpus and prepyloric regions. Hypervolemia also shows the highest oxygenation levels in the corpus. There were no statistically significant changes between the measurement times (hypotension, normotension, and hypertension, respectively).

The local tissue oxygenation in the control group showed significantly decreasing values from the prepyloric region to the tip. In group 2 area D1 had a significantly decreased oxygenation compared to D2 and D3. Application of volume led to the highest tissue oxygenation in area D2. There were no significant changes within an area at the different measurement times. The tissue oxygenation of all groups is shown as box plots in Fig. 7.

### Assessment of organ perfusion by FM

The distal part of gastric tube D1 (T1: 1.04 ± 0.45 mL/min/g; T2: 0.92 ± 0.23 mL/min/g; T3: 1.12 ± 0.45 mL/min/g) showed impaired perfusion compared to the proximal parts (D3/D2). Increasing MAP was associated with increasing perfusion in area D3 (T1: 1.12 ± 0.41 mL/min/g; T2: 1.20 ± 0.35 mL/min/g; T3: 1.45 ± 0.37 mL/min/g). The gastric corpus (D2) showed similar results (T1: 1.12 ± 0.46 mL/min/g; T2: 1.14 ± 0.31 mL/min/g; T3: 1.51 ± 0.59 mL/min/g).

Group 2 showed reduced flow in all areas and all measurements compared to the control group. At prepyloric region D3, increasing MAP resulted in higher perfusion (T1: 0.61 ± 0.22 mL/min/g; T2: 0.74 ± 0.41 mL/min/g; T3: 0.92 ± 0.33 mL/min/g). In area D2, there were slight changes in perfusion between hypotension and normotension (T1: 0.65 ± 0.29 mL/min/g; T2: 0.77 ± 0.46 mL/min/g; T3: 0.77 ± 0.36 mL/min/g). Area D1 showed similar perfusion levels (T1: 0.60 ± 0.31 mL/min/g; T2: 0.76 ± 0.63 mL/min/g; T3: 0.74 ± 0.45 mL/min/g).

The perfusion in group 3 was also impaired in all areas and measurements compared to the control group. Perfusion in the prepyloric region showed the highest levels at normotension (T1: 0.34 ± 0.09 mL/min/g; T2: 0.92 ± 0.33 mL/min/g; T3: 0.51 ± 0.26 mL/min/g). Area D2 (T1: 0.51 ± 0.23 mL/min/g; T2: 0.79 ± 0.47 mL/min/g; T3: 0.70 ± 0.44 mL/min/g) and D1 (T1: 0.39 ± 0.21 mL/min/g; T2: 0.74 ± 0.36 mL/min/g; T3: 0.52 ± 0.32 mL/min/g) showed similar trends.

### Histology

Indicating impaired perfusion, histological findings showed loss of epithelium towards the tip of the gastric tube. Compared to the control group (D1 1.48; D2 0.76; D3 0.33), the degree of damage washigher in the nitroglycerine (D1 1.52; D2 1.52; D3 0.83) and volume group (D1 1.33; D2 1.00; D3 0.76), particularly in the prepyloric region and corpus.

## Discussion

Anastomotic leakage remains a life-threatening complication after esophagectomy^24^. Due to the singular vascular supply of the gastric tube, impaired perfusion should be emphasized as an independent risk factor^25^. Up to now, various techniques for intraoperative measurement of local tissue perfusion have been described^26,27^. Among these, ICG-FI is a promising tool for a real-time, non-invasive assessment of gastric tube perfusion^28^. Several retrospective studies using ICG-FI during esophagectomy have reported a reduction of the anastomotic leakage rate of up to 69% compared to standard-of-care procedures^29–33^. Previously, we validated the quantification of ICG-FI by using fluorescent microspheres as the gold standard for experimental perfusion assessment. We were the first to show that ICG-FI can sufficiently predict the local tissue perfusion of the gastric tube by using the described quantification parameters^7^. Moreover, in an additional study on intraoperative evaluation of gastric tube perfusion in patients undergoing esophagectomy we quantified the perfusion using SFI, BSFI and TTS. In fact, perfusion reduction of up to 32% was not associated with anastomotic leakage^20^. The present study aimed was to investigate the influence of hypervolemia and continuous administration of nitroglycerine on gastric tube perfusion quantified by ICG-FI and FM.

In our recent study, we objectified two key aspects of gastric tube microperfusion. By establishing three different settings of hemodynamic management according to different intraoperative settings, we have observed the influence of hypervolemia and continuous administration of nitroglycerine on the gastric tube perfusion. FM, as the gold standard of experimental tissue perfusion measurement, demonstrated a significantly reduced microperfusion of the whole gastric tube in the setting of hypervolemia or after administration of nitroglycerine compared to the control group treated with catecholamines. In addition, there was a loss of hemodynamic coherence resulting in increased systemic MAP without improving the gastric tube microperfusion. Furthermore, hypervolemia was associated with a reduction of perfusion level compared to normotension, whereas administration of nitroglycerine resulted in no significant changes in microperfusion levels with increasing MAP. TTFM showed a comparable pattern of flow changes, which, however, were not statistically significant. The local tpO_2_ was consistent with the FM results of group 2 without significant changes of microperfusion between the measurements. However, the perfusion changes in group 3 could not be detected by tPO_2_ measurement. Therefore, TTFM and tPO_2_ are inappropriate for the evaluation of microperfusion. Moreover, histopathological findings supported these results with a higher degree of epithelial damage after administration of nitroglycerine or volume, especially in the corpus and at the tip of the gastric tube.

In addition, ICG-FI has been validated as a suitable technology for evaluating gastric tube microperfusion by confirming the FM results. While MAP as the standard parameter for intraoperative and postoperative hemodynamic management was comparable in all groups at the different measurements, ICG-FI, especially SFI, was able to confirm the changes of microperfusion not only between different MAP levels, but also between the groups with different treatment at identical MAP. Furthermore, SFI and BSFI correlated significantly with FM results underlining the consistency of the two methods for evaluating microperfusion. Even though FM represents the gold standard in experimental microperfusion measurement, it remains a technically non-trivial, invasive, and, in terms of instrumentation and interpretation, complex and time-consuming method. The perfusion evaluation with FM is only suitable for experimental animal settings. In comparison, ICG-FI is a non-invasive technology, which can be evaluated, and interpreted immediately. In addition, ICG-FI can be used clinically on humans to verify animal experimental results.

In contrast to BSFI and SFI, TTS-ratio could only reliably show the perfusion changes within the respective groups. This is mainly due to the calculation of the ratio to the prepyloric region as the baseline, since the delay in the first fluorescence signal between the groups was not considered.

Several strategies for perioperative hemodynamic management have been published. The use of catecholamines as a standard method for perioperative blood pressure management and their influence on the perfusion of the gastric tube are controversially discussed. Theodorou et al. observed an increasing blood lactate level after hemorrhagic shock and consecutive administration of norepinephrine in an experimental porcine model. They concluded that vasoconstriction reduced the gastric tube microperfusion^34^. In contrast, Al-Rawi et al. demonstrated a sufficient increase of microcirculation of the gastric tube after administration of catecholamine in 12 patients^35^. Moreover, in a recently published retrospective analysis of 494 patients undergoing esophagectomy, intraoperative vasopressor use was not associated with anastomotic leakage^36^. Our results support the use of catecholamine administration for hemodynamic management in patients undergoing esophagectomy and reconstruction with gastric tube pull-up. The control group showed significantly better perfusion levels, and increasing MAP was associated with perfusion improvement.

As a hypothesis, nitroglycerine as a vasodilator agent might reduce venous congestion in the gastric tube. In a porcine model, van Bommel et al. investigated the effect of continuous application of nitroglycerine during gastric tube formation. Nitroglycerine could improve the microperfusion in the corpus and fundus region compared to a control group^37^. However, in a prospective, randomized, controlled trial, nitroglycerine was administrated intravenously during gastric tube reconstruction in patients undergoing an esophagectomy. There was no significant difference regarding anastomotic leakage rate, microvascular blood flow, and microvascular hemoglobin oxygen saturation compared to the control group^38^. Our findings are partially in line with those of Buise et al. by describing no improvement of microperfusion by administration of nitroglycerine. However, in contrast to their results, we could demonstrate that a continuous administration of nitroglycerine was even associated with impaired perfusion at all measurement times compared to the control group.

Perioperative volume management during major surgery affects the surgical outcome^39^. In several clinical studies, hypervolemia was associated with increased rates of anastomotic leakage^40–43^. Moreover, in a prospective clinical trial comparing liberal fluid management with goal-directed fluid therapy in patients undergoing pancreaticoduodenectomy visceral tissue edema measured by CT-scan could be identified as an independent risk factor for severe surgical complications^44^. Furthermore, hypervolemia could be identified as an independent risk factor for higher postoperative morbidity in patients undergoing esophagectomy^41^. Supporting those already published data, we could demonstrate that hypervolemia is associated with significantly reduced microperfusion of the gastric tube using ICG-FI for quantification. Increased morbidity, especially anastomotic leakage, can be explained by impaired microperfusion being an independent risk factor for anastomotic leakage.

Due to the large experimental animal setting, results should be interpreted with caution regarding translation into humans. Another limitation is the small cohort size due to the smallest necessary number of test animals.

Based on our results, we conclude that hypervolemia and continuous application of nitroglycerine are associated with impaired microperfusion of the gastric tube as measured by ICG-FI and FM compared to hemodynamic management using norepinephrine. Our results support current clinical practice with restrictive volume and catecholamine administration in major surgery. Hypervolemia should be avoided in esophagectomy with gastric tube pull-up. Furthermore, continuous administration of nitroglycerine is not suitable for improving microperfusion of the gastric tube. In addition, an evaluation and valid quantification of gastric tube microperfusion using ICG-FI is possible by determining SFI and BSFI. The smallest differences in microperfusion could be sufficiently predicted, whichcould not be represented by the MAP measurement. However, further validation of ICG-FI with prospective clinical trials and survival studies is needed.

## Data Availability

The datasets used and analyzed during the current study are available from the corresponding author on reasonable request.
